# Intervention effects of low-molecular-weight chondroitin sulfate from the nasal cartilage of yellow cattle on lipopolysaccharide-induced behavioral disorders: regulation of the microbiome-gut-brain axis

**DOI:** 10.3389/fnut.2024.1371691

**Published:** 2024-05-21

**Authors:** Yuxuan Deng, Qingyuan Li, Junxian Song, Rui Guo, Tianchen Ma, Zhigang Liu, Qian Liu

**Affiliations:** ^1^College of Food Science and Technology, Northwest University, Xi’an, China; ^2^Laboratory of Functional Chemistry and Nutrition of Food, College of Food Science and Engineering, Northwest A&F University, Yangling, Shaanxi, China

**Keywords:** chondroitin sulfate, oxidation degradation, anti-inflammatory, cognitive function, intestinal microbiota

## Abstract

Chondroitin sulfate (CS) is a sulfated linear polysaccharide with different functional activities, including antioxidant, anti-inflammatory, lipid-lowering, and immune regulation. As natural sulfated polysaccharides have high molecular weight, high apparent viscosity, low water solubility, complex structure, and high negative charge, they have difficulty binding to receptors within cells across tissue barriers, resulting in low bioavailability and unclear structure–activity relationships. In this study, an H_2_O_2_-Vc oxidative degradation system was employed to perform environmentally friendly and controllable degradation of CS extracted from the nasal cartilage of Shaanxi Yellow cattle. Two low-molecular-weight chondroitin sulfates (LMWCSs), CS-1 (14.8 kDa) and CS-2 (50.9 kDa), that exhibit strong *in vitro* free radical scavenging ability were obtained, and their structures were characterized. Mice intraperitoneally administered lipopolysaccharide (LPS) were used to explore the cognitive intervention effects of LMWCS. Supplementing CS-1 and CS-2 significantly downregulated the levels of the serum inflammatory factors, TNF-α and IL-1β, promoted the expression of GSH in the brain, and inhibited the production of the lipid peroxidation product, malondialdehyde (MDA), ultimately inhibiting LPS-induced cognitive impairment in mice. Surprisingly, compared to the LPS model group, the abundances of *Streptococcus*, *Eisenbergiella*, *Vampirovibrio*, *Coprococcus*, *Enterococcus* and *Lachnoanaerobaculum* were significantly increased in the intestines of mice in the CS-1 and CS-2 group, whereas those of *Parabacteroides* and *Mycoplasma* were significantly decreased. Altogether, this study provides a theoretical basis for the comprehensive utilization of agricultural and animal resources and the application of brain nutrition, anti-inflammatory, and LMWCS health products.

## 1 Introduction

Inflammation is the coping mechanism of innate immune system to resist external injury and protect the body. The occurrence of inflammation is to control and remove external stimuli, activate inflammasome and phagocytosis, even trigger apoptosis, and finally promote tissue regeneration. Although the inflammatory response is originally intended to protect the body, over-generated inflammatory factors can also cause tissue damage and diseases. Peripheral inflammation can trigger neuroinflammation, in which the blood–brain barrier (BBB), glial cells and neuronal cells play indispensable roles (1). The BBB is a highly specialized endothelial tissue used to isolate the central nervous system (CNS) and the peripheral immune system. However, this endothelial membrane not only allows the passage of proinflammatory mediators produced by peripheral inflammatory responses, but also allows leukocytes to exercise them into the brain. This will trigger synaptic damage and neuronal apoptosis, and intensify a variety of brain neuropathy. At the same time, a series of inflammatory cytokines such as tumor necrosis factor-α (TNF-α), interleukin-1β (IL-1β), and interleukin-6 (IL-6) will also destroy the integrity of BBB, increase its permeability, and further promote the migration of leukocytes to the brain (2). The persistent chronic neuroinflammation can also promote the production of reactive oxygen species, increase oxidative stress and the deterioration of neuronal damage, thus destroying the function of CNS (3). Neuroinflammation is the core pathogenesis and one of the main early pathological features of mild cognitive impairment (4). In addition, according to numerous studies, high levels of neuroinflammation are closely related to emotional disorders, such as anxiety, depression, and addiction (5).

The gut-brain has a two-way regulatory function in nerve, endocrine, immunity, and metabolism, and is related to inflammatory bowel disease, obesity, diabetes, insulin resistance, and other gastrointestinal and metabolic diseases (6). Microorganisms that colonize the intestine play an indispensable role in brain and gut communication. This communication occurs through pathways, including the vagus nerve, gut hormone signaling, immune system, tryptophan metabolism, and microbial metabolites, such as short chain fatty acids (SCFAs), namely the gut microbiota-gut-brain axis (MGBA) (7). In clinical trials, Vogt et al. observed significant changes in the gut microbial diversity, such as a decrease in *Firmicutes* and *Bifidobacteria* in the gut and an increase in *Bacteroides*, in patients with Alzheimer’s disease (AD) (8). An increase in the abundance of a pro-inflammatory taxon, *Escherichia/Shigella*, and a reduction in the abundance of an anti-inflammatory taxon, *E. rectale*, are possibly associated with a peripheral inflammatory state in patients with cognitive impairment and brain amyloidosis (9). Lipopolysaccharide (LPS) produced by bacteria has been demonstrated can induce systemic inflammation, which leads to the increase of proinflammatory cytokines. Furthermore, the central nervous is also impacted by these cytokines, showing the formation of cortical amyloid proteins (10). These changes will trigger cascade immune responses and contribute to the development of neurodegenerative diseases (11). According to Kim et al., high-fat diet mice exhibited enhanced systemic and central nervous system inflammatory responses, which affected MGBA, resulting in reduced cognitive function and increased β-amyloid protein accumulation, with a significantly changed gut microbiome (12). Therefore, improving gut microbiota homeostasis may offer an effective approach to suppress neuroinflammation, and consequently, enhance cognitive function.

Chondroitin sulfate (CS) is a sulfated linear polysaccharide mainly composed of a disaccharide unit consisting of [→4)- β- D-GlcA – (1 → 3)- β- D-GalNAc – (1→], and is widely distributed in tissues, including the cartilage, umbilical cord, and vascular wall of humans and certain animals, such as pigs, cows, sheep, and fish (13). CS has a range of vital biological functions, including antioxidant, anti-inflammatory, lipid-lowering, and immunomodulatory activities, and is listed as a leading therapeutic drug for the prevention and treatment of cardiovascular and joint diseases (14–17). Therefore, the identification of new sustainable CS resources is of great significance. As natural sulfated polysaccharides have high molecular weight, high apparent viscosity, low water solubility, complex structure, and high negative charges, binding to cellular receptors is a difficult challenge owing to tissue barriers, resulting in low bioavailability and unclear structure–activity relationships. Baici et al. revealed that complete chain-length CS has difficulty penetrating the gastric and intestinal mucosa, whereas low-molecular-weight chondroitin sulfate (LMWCS) can penetrate the intestinal mucosa (18). Our research group extracted CS from the bovine nasal cartilage in the initial stage and used H_2_O_2_-Vc as a degradation system to obtain four LMWCS variants. The degradation component with the lowest molecular weight (19.7 kDa) had the strongest antioxidant activity *in vitro* (19). However, the structure–activity relationship between CS and LMWCS in improving neuroinflammation remains unclear, and the specific mechanisms by which they exert cognitive intervention, especially the regulatory effects of MGBA, require further in-depth investigation.

This study used cost-effective Shaanxi agricultural and animal husbandry by-products, particularly yellow cattle nasal cartilage, as the main raw material. LMWCS was obtained via controllable degradation of purified components using low consumption, ecological, and industrial-scale production oxidation degradation methods, and their structures were characterized. By intraperitoneally administering LPS to induce neuroinflammation mouse model, this study aimed to explore the cognitive intervention mechanism of LMWCS extracted from the nasal cartilage of yellow cattle based on MGBA to achieve high-value use of yellow cattle and contribute to the theoretical basis for the comprehensive exploitation of agricultural and animal resources, and the development of anti-inflammatory LMWCS health products for brain nutrition.

## 2 Materials and methods

### 2.1 Materials and chemicals

Yellow cattle nose cartilage was procured from Xi’an Huimin Street (Shanxi, China). Chondroitin sulfate standard (CAS 9007-28-7, purity 95%) was obtained from Macklin Company (Shanghai, China). H_2_O_2_ and Vc were purchased from Chengdu Kelong Chemical Co., Ltd. (Chengdu, China). Glucan standards (1, 5, 12, 25, 50, 80, 150, and 270 kDa) were obtained from Sigma Aldrich (St. Louis, MO, United States). Lipopolysaccharide (LPS) was purchased from Sigma Aldrich (St. Louis, MO, United States); FeSO_4_ and FeCl_3_·6H_2_O were purchased from Tianjin Damao Chemical Reagent Factory. 2,3,5-Chlorotriphenyltetrazolium (TTC) was obtained from Shanghai Lanji Technology Development Co., Ltd. Enzyme-linked immunosorbent assay (ELISA) kits for IL-1β and TNF-α were purchased from Shanghai Xinle Biotechnology Co., Ltd. (Shanghai, China). Malondialdehyde (MDA) and reduced glutathione (GSH) detection kits were obtained from Nanjing Jiancheng Biotechnology Research Institute. Acetic acid, propionic acid, butyric acid, isobutyric acid, valeric acid, and n-valeric acid standards were purchased from Aladdin Bio-Chem Technology Co., Ltd. (Shanghai, China). BIOZOL reagent was purchased from Hangzhou Bioer Technology Co., Ltd. (Zhejiang, China). Reverse Transcription Kit (UEIris RT mix with DNase) and 2× SYBR Green qPCR Master Mix were purchased from Suzhou Hengyu Biotechnology Co., Ltd. (Jiangsu, China). All other reagents used in this study were manufactured in China and met the standards of high-performance liquid chromatography (HPLC) or the highest commercially available grade.

### 2.2 Extraction and purification of CS from bovine nasal cartilage

The meat and impurities attached to the surface of the bovine nasal cartilage were removed, washed, degreased with acetone, and air dried. Thereafter, 10 g of bovine nasal cartilage powder was weighed and suspended in 0.01 M EDTA buffer containing 0.01 M cysteine (in a ratio of 1:10, w/v) with pH adjusted to 6.5. A 1.5% composite enzyme solution (papain: trypsin = 1:2, w/w) was added to the mixture and allowed to react in a 65°C water bath for 22 h. Following centrifugation at 4000 rpm for 10 min, the supernatant was collected and precipitated with 1.25 volume of ethanol at 4°C for 12 h, followed by 2 volumes of ethanol containing 0.5% sodium acetate. The resulting solution was centrifuged at 4000 rpm for 10 min, and the precipitate was dissolved in 200 mL of 0.5 M NaCl solution containing 5% cetylpyridinium chloride (CPC). After centrifugation at 4000 rpm for 10 min, the precipitate was dissolved in 100 mL of 2 M NaCl: ethanol (100:15, v/v) and precipitated with three volumes of ethanol. The above steps were repeated, and the obtained sediment was centrifuged, dissolved in water, and then dialyzed against ultrapure water for 3 days. The solution was concentrated under reduced pressure and freeze-dried to obtain the CS samples.

### 2.3 Screening of the oxidative degradation conditions for CS

To obtain components with different molecular weights, degradation conditions were screened using a controlled variable method. Briefly, 50 mg of CS was weighed accurately for each serving, and then suspensions were prepared with a material liquid ratio of 0.5% and transferred into stoppered vials. The concentration of H_2_O_2_ was maintained at 60 mM and that of Vc was changed from 0.5 mM to 60 mM. Conversely, the concentration of Vc was maintained at 20 mM and the concentration of H_2_O_2_ was changed from 0.5 mM to 80 mM. After sealing, the solutions were allowed to react for 3 h in a 60°C water bath. Upon reaction completion, the solution was concentrated, dialyzed at 4°C for 3 days using a dialysis bag with a molecular cut-off of 3,500 Da (excluding H_2_O_2_ and Vc), and freeze-dried to collect the degraded samples.

### 2.4 Physicochemical properties of LMWCS

The carbazole sulfuric acid method was used to determine the glucuronic acid content (20). Using potassium sulfate as the standard, the barium chloride gel method was used to measure the sulfate content (21). Protein content was determined using the BCA kit method (22). The FT-IR spectra (KBr particles) of the polysaccharides (2 mg) were recorded in the range of 400–4,000 cm^−1^ using a Nicolet 5,700 spectrometer (Thermo Fish Scientific Inc. Waltham, MA, United States). The average molecular weights of CS and its degradation components were determined using HPLC with a TSK Gel G4000SWXL dextran gel chromatographic column, differential refractive detector, mobile phase consisting of phosphate buffer brine with pH 6.0, flow rate of 0.3 mL/min, and injection volume of 20 μL.

### 2.5 Antioxidant activities determination of LMWCS *in vitro*

The ability of CS and its degradation products to remove ABTS^+^· was determined using the method of Li et al. (23). The scavenging activity of 1-diphenyl-2-picrylhydrazine (DPPḤ) was determined using the Yang et al. method (24). The Fenton method was used to determine the scavenging potential of CS and its degradation products on hydroxyl radicals (19). The abilities of CS and its degradation products to reduce iron ions were measured using the method described by Unver et al. (25).

### 2.6 Animals and treatment

The 3-month-old C57BL/6 J mice were obtained from Xi’an Jiaotong University (Xi’an, Shaanxi, China). Mice were housed in an animal facility under standard environmental conditions (12/12 h light dark cycle, humidity 50 ± 15%, temperature 22 ± 2°C) and were provided with a standard feed (AIN-93 M). Mice were randomly divided into four groups: CON, LPS, CS-1 + LPS, and CS-2 + LPS (*n* = 10/group). CS-1 + LPS and CS-2 + LPS group mice were administered CS-1 or CS-2 (200 mg/kg/d, dissolved in physiological saline) via oral gavage over 64 consecutive days. Mice in the LPS, CS-1 + LPS, and CS-2 + LPS groups were intraperitoneally administered LPS (0.25 mg/kg/d, dissolved in physiological saline), while those in the CON group received an equivalent volume of physiological saline for 18 consecutive days. This dosage of LPS was chosen based on previous studies (26, 27). At the end of the experiment, the mice were then placed in an induction chamber and exposed to 4% isoflurane-oxygen mixture until they were unconscious, as confirmed by the lack of a pedal withdrawal reflex. Then, the mice were euthanized through cervical dislocation, after which both serum and brain samples were collected, and stored at −80°C for further analysis. The animal protocol was approved by the Animal Ethics Committee of Northwest A&F University. All experimental procedures were performed in strict accordance with the “Guidelines for the Care and Use of Experimental Animals” (8th edition; ISBN-10:0-309-15396-4).

### 2.7 Animal behavioral experiments

#### 2.7.1 Y-maze test

After intraperitoneal injection of LPS for 4 h, the working memory ability of mice was evaluated using a Y-maze experiment. The Y-maze consisted of three identical arms, each at a 120-degree angle and measuring 35 × 5 × 15 cm (length × width × height). A single mouse was placed at the center of the Y-maze and allowed to freely explore the maze for 8 min. Successful alternation was defined as consecutive entries into a new arm before returning to the two previously visited arms. The total number of arm attempts and the number of consecutive arm attempts were recorded by a video analysis system, and the percentage of alternation was calculated using the following formula:


$$\mathrm{Spontaneous}\;\mathrm{alternation}\;\left(\%\right)=\frac{\begin{array}{l} \mathrm{Number}\;\mathrm{of}\;\\ \mathrm{alternations} \end{array}}{\begin{array}{l} \mathrm{Number}\;\mathrm{of}\;\\ \mathrm{total}\;\mathrm{armentries}-2 \end{array}}\times 100$$

#### 2.7.2 Novel object recognition test

Using the innate tendency of mice to explore novel objects, a new object recognition test was conducted to evaluate the long-term memory and learning ability of mice under stress-free conditions. Before each test, 75% alcohol was used to remove odors and clean the test device. On the first day of the experiment, mice were placed in a 40 × 40 × 40 cm acrylic box to adapt to the experimental device. On day 2 of the experiment, two identical odorless objects were placed in front of each mouse. Mice freely explored the objects for 10 min, and the exploration time was recorded. On day 3, an old object (used on the second day of the experiment) and a novel object were placed in the same position. Mice were allowed to explore freely for 5 min, and their activity was recorded and archived using the SuperMaze video analysis system. The time taken by mice to explore new and old objects, and the total time taken to explore these objects, were recorded, and the new object recognition index was calculated according to the following formula:


$$\mathrm{Discrimination}\;\mathrm{index}=\frac{\begin{array}{l} \mathrm{New}\;\mathrm{object}\;\mathrm{exploration}\;\mathrm{time}\\ -\mathrm{Old}\;\mathrm{object}\;\mathrm{exploration}\;\mathrm{time} \end{array}}{\mathrm{Total}\;\mathrm{exploration}\;\mathrm{time}}$$

#### 2.7.3 Barnes maze test

The Barnes maze test was performed as previously described (28). The maze consists of a circular platform (r = 50 cm) surrounded by 20 small holes (r = 5 cm) that penetrate the platform. An escaping box was placed at the bottom of one of the holes as a shelter for the mouse, and the other holes were empty. The bright light was used as the motivation for the mouse to enter the escaping box. The test was completed over 5 days. Adaptation phase (day 0): Before the start of the experiment, the mouse was placed in the avoidance box (escape room), which was connected to the target hole, to adapt for 60 s first. Subsequently, the mouse was held in the black activation box at the center of the maze to limit its range of movement for another 60 s to reduce the anxiety and exploratory behavior before the training began. Acquisition phase (days 1–3): The 180 s learning trial was conducted. The mice were initially placed within the black activation box at the center of the maze to restrict their activity for a duration of 5 s, and then the activation box as moved away to allow the mice to find the escape room. The experimenter observed their activities through a camera in another room. If a mouse enters the escape room with all its limbs, it is considered a successful escape. Regardless of whether the mouse located the target hole or entered the box, it was placed in the escaping box for another 60 s. After each test, mouse feces were cleaned, and the disk surface was wiped with alcohol to eliminate odors. Additionally, the upper disk was rotated randomly before each training session without changing the location of the escape room. The purpose is to prevent mice from relying on smell rather than memory to locate escape rooms. Each mouse is trained once a day for 3 days, intended to strengthen the animals’ spatial learning and memory capabilities through repetitive training. Testing phase (day 4): The escaping box was removed, and the mouse was positioned within the black box at the center of the maze for 5 s, then allowed to explore freely for 180 s. The time for first finding the target hole and the number of exploring the wrong hole were recorded by a video tracking system (SuperMaze software, Shanghai Xinruan Information Technology Co., Ltd., China). At the end of each mouse experiment, the platform and escaping box were cleaned with 75% ethanol to eliminate the smell.

### 2.8 Hematoxylin-eosin staining

Mouse brain tissue was fixed in 4% (v/v) paraformaldehyde and embedded in paraffin. Brain slices were cut into 5 mm slices using a slicer. Before staining, sections were dewaxed with xylene and rehydrated with each gradient ethanol (100, 90, 80, and 70%) for 5 min, and washed five times with PBS (pH 7.4) for 5 min each. The brain slides were stained using H&E.

### 2.9 Detection of inflammatory factors and antioxidant activities

Commercial ELISA kits (Mouse TNF- α Reagent kit, IL-1 β test kit, Shanghai Xinle Biotechnology Co., Ltd., China) were used to determine the levels of TNF-α and IL-1β in mouse serum. The levels of MDA and GSH in the brain tissue were measured according to the instructions of the Nanjing Jiancheng kits.

### 2.10 RNA extraction and real-time quantitative PCR

Total RNA was extracted from the brain tissue using the BIOZOL reagent (Hangzhou Bioer Technology Co., Zhejiang, China). RNA concentrations were determined using a NanoDrop 2000/2000C (Thermo Fisher Scientific; Waltham, MA, United States), and diluted to the same concentration with enzyme-free water. RNA was reverse transcribed into cDNA using the cDNA synthesis kit (US Everbright, China) according to the manufacturer’s protocol. After reverse transcription, the samples were diluted 5-fold with enzyme-free water, and then the reaction system was prepared with the 2× SYBR Green qPCR Master Mix (Suzhou Hengyu Biotechnology Co., Ltd., Jiangsu, China) for quantitative analysis in a LightCycler^®^ 96 (Roche Diagnostics, Switzerland) using the following condition: 95°C for 120 s, followed by 40 cycles of 95°C for 5 s and 60°C for 30 s. The mouse primers are listed in Table 1. *Gapdh* was used as an internal reference and relative gene expression was calculated using 2 ^− △△Ct^.

**Table 1:** Primer sequences for semi quantitative RT-PCR analysis.

	Forward primer	Reverse primer
*Il-1β*	TGACGGACCCCAAAAGATGA	TCTCCACAGCCACAATGAGT
*Tnf-α*	CCCTCACACTCAGATCATCTTCT	GCTACGACGTGGGCTACAG
*Gapdh*	TGGAGAAACCTGCCAAGTATGA	TGGAAGAATGGGAGTTGCTGT

### 2.11 16S rRNA sequencing and intestinal microbiota analysis

Microbial community analysis was performed by Shenzhen Huada Gene Technology Service Co., Ltd. (Shenzhen, China). The total DNA of the microbial community was isolated from mouse fecal samples according to the E.Z.N.A. Stool DNA Extraction Kit (Omega Biotek, Norcross, GA, United States). The V3–V4 hypervariable regions of the bacteria 16S rRNA gene were amplified with primers 341F (5’-CCTACGGGNGGCWGCAG-3′) and 806R (5’-GGACTACHVGGGTATCTAAT-3′) by thermocycler PCR system (GeneAmp 9,700, ABI, United States). The PCR reactions were conducted using the following program: 3 min of denaturation at 95°C, 27 cycles of 30 s at 95°C, the 30 s for annealing at 55°C, and 45 s for elongation at 72°C, and a final extension at 72°C for 10 min. PCR reactions were performed in triplicate 20 μL mixture containing 4 μL of 5 × FastPfu Buffer, 2 μL of 2.5 mM dNTPs, 0.8 μL of each primer (5 μM), 0.4 μL of FastPfu Polymerase and 10 ng of template DNA. Genomic DNA was randomly fragmented, which will be selected by magnetic beads to purify PCR-amplified products and dissolved in an elution buffer to complete library construction. The fragment range and concentration of the library were checked using the Agilent 2,100 Bioanalyzer. Qualified libraries were sequenced on the DNBSEQ platform by MGI2000 according to the size of the inserted fragments. The filtered clean data were used for later analysis. Sequence splicing was performed using FLASH software (Fast Length Adjustment of Short reads, v1.2.11), and the paired reads were spliced into a sequence through overlapping relationships to obtain tags of hypervariable regions. The software USEARCH (v7.0.1090) was used to cluster the spliced tags into OTUs (Operational Taxonomic Units), compared with the database, and species annotation. Based on the OTU and annotation results, species difference analyses between groups were performed by the Kruskal test. R (version 3.1.1) with the “vegan” package was employed to conduct the Principal Coordinate Analysis (PCoA), and the “ggplot2” package was utilized for the visualization of the PCoA diagram. R (v3.1.1) “gplots” package was used to form a heatmap. R (v3.4.1) was used to make species histograms.

### 2.12 Detection of short chain fatty acids

As previously described (29), the concentration of SCFAs in feces were detected using gas chromatography (GC). The standard curve was constructed with the standard mixture of different concentrations of SCFAs. Briefly, 0.20 g feces were homogenized with 1 mL of MilliQ water and 0.15 mL 50% H_2_SO_4_ (w/w). The mixture was added with 1.6 mL of diethyl ether, and the samples were incubated on ice for 20 min. Centrifuge at 8000 rpm for 5 min to obtain supernatant, and filter through 0.2 μm filter (Branch billion Lung Experimental Equipment Co., Ltd., Tianjin, China) into clear GC vials. The standards of SCFAs included acetate, A116173; propionate, P110445; butyrate, B11se0438 (Aladdin Bio-Chem Technology Co., LTD, Shanghai, China). The concentration of SCFAs in fecal was analyzed using a GC-2014C gas chromatograph (Shimadzu Corporation, Kyoto, Japan), equipped with a DB-FFAP capillary column (Agilent Technologies, Wilmington, DE, United States) and flame ionization detector. The following heating program: initial temperature was employed: initially 50°C for 3 min, 10°C/min to 130°C, 5 ° C/min to 170°C, 15 ° C/min to 220°C, where it was maintained with maintenance for 3 min. The injector temperature was 250°C and the detector temperature was 270°C.

### 2.13 Data analysis

*In vivo* data are presented as mean ± SEM of at least six independent experiments, and *in vitro* data are presented as mean ± SD. Significant differences among means were evaluated using one-way ANOVA followed by the Tukey post-hoc test for multiple comparisons in GraphPad Prism 8.0 software (GraphPad Software Inc., San Diego, CA, United States). A *p*-value of less than 0.05 was considered statistically significant.

## 3 Results

### 3.1 Preparation and physicochemical properties of LMWCS

CS was extracted from the nasal cartilage of Shaanxi yellow cattle using a complex enzyme method (papain: trypsin = 1:2). The resulting CS sample appeared as a white powder, with yield of 7.50 ± 0.34%. In this study, CS degradation was achieved using the H_2_O_2_-Vc oxidation method; this method was selected owing to its mild degradation conditions and simplicity, resulting in minimal structural damage to the glycan chains. By changing the concentrations of H_2_O_2_ and Vc, CS components with different molecular weights were prepared.

**Figure 1:**
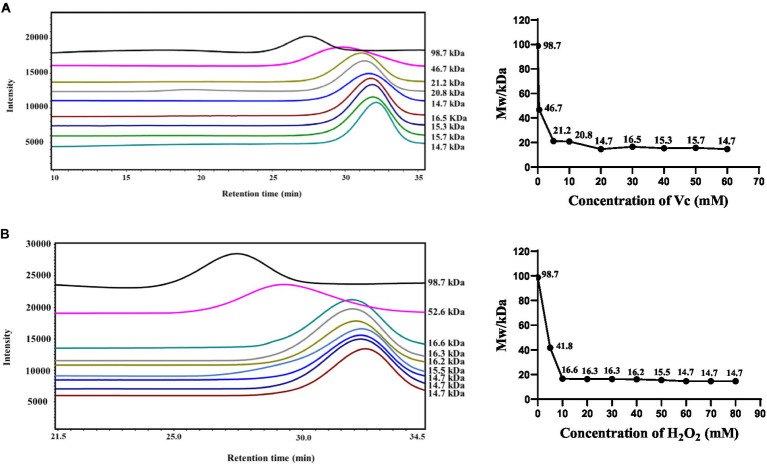
Effect of the H_2_O_2_/Vc degradation system on the molecular weight of CS. **(A)** Effect of Vc concentration on the molecular weight of CS; **(B)** Effect of H_2_O_2_ concentration on the molecular weight of CS.

Maintaining the concentration of H_2_O_2_ (60 mM) and varying the concentration of Vc (0–60 mM), as shown in Figure 1A, resulted in a gradual decrease in the molecular weight of the sample with increasing Vc concentration. When the concentration of H_2_O_2_ was 60 mM and that of Vc was 20 mM, the degree of degradation reached its maximum, and the molecular weight decreased to 14.7 kDa. The concentration of Vc (20 mM) was maintained while that of H_2_O_2_ was varied from 0 to 80 mM, as illustrated in Figure 1B. At an H_2_O_2_ concentration of 40 mM, the molecular weight decreased to 16.2 kDa. With a further increase in the concentration of H_2_O_2_, the molecular weight stabilized at approximately 15 kDa. When the concentration of H_2_O_2_ was increased from 60 to 80 mM, the change in molecular weight was not significant, possibly because the concentration of H_2_O_2_ reached an optimal range.

Since the molecular weight of CS before degradation was 98.7 kDa, in order to further explore the effects of molecular weight on the biological activity of CS, we prepared medium molecular weight segments (CS-2, 50.9 kDa), which produced when the concentration of H_2_O_2_ was 7 mM and Vc was 7 mM. In addition, the commercially available CS standard (CS-S, 80.8 kDa) was used as a control for subsequent studies. The physicochemical properties of CS, CS-S, and their degradation components, CS-1 and CS-2, are shown in Table 2. No significant changes were found in the sulfate and uronic acid contents of the degraded CS-1 and CS-2, and CS.

**Table 2:** Physicochemical properties of CS and its degradation components.

Sample	Uronic acid content (%)	Protein content (%)	Sulfate content (%)	Mw (kDa)
CS-S	32.72 ± 1.04 ^a^	1.14 ± 0.10 ^c^	22.07 ± 1.20 ^b^	80,812
CS	35.72 ± 1.36 ^a^	2.05 ± 0.13 ^b^	22.59 ± 0.45 ^ab^	98,699
CS-1	33.11 ± 3.47 ^a^	3.61 ± 0.17 ^a^	22.17 ± 1.14 ^ab^	14,691
CS-2	31.57 ± 1.89 ^a^	3.80 ± 0.26 ^a^	24.11 ± 1.05 ^a^	50,949

Different letters indicated significant differences between the components, *p* < 0.05.

Fourier transform infrared (FT-IR) spectroscopy is a valuable tool for assessing the structure of polysaccharides and obtaining data on their functional groups and chemical bonds. FT-IR spectroscopy was conducted using CS and its degradation components within the range of 4,000 cm^−1^ to 500 cm^−1^ (Supplementary Figure S1). The peaks around 1,238 cm^−1^ and 856 cm^−1^ were derived from the tensile vibration of sulfate S=O in the axial position and the bending vibration of C-O-S, respectively, indicating the presence of several spectral bands associated with sulfate esters. The signals of O-H stretching vibration and C=O asymmetric stretching vibration of N-acetylaminogalactose and glucuronic acid were visible at 3441 cm^−1^ and 1,639 cm^−1^. Furthermore, the symmetric stretching vibration of glucuronic acid COO^−^ and the stretching vibration of C-O within COOH were identified at 1410 cm^−1^ (30). These results indicate that H_2_O_2_ and Vc did not damage the main functional groups involved in the degradation of polysaccharides.

### 3.2 *In vitro* antioxidant activities of LMWCS

**Figure 2:**
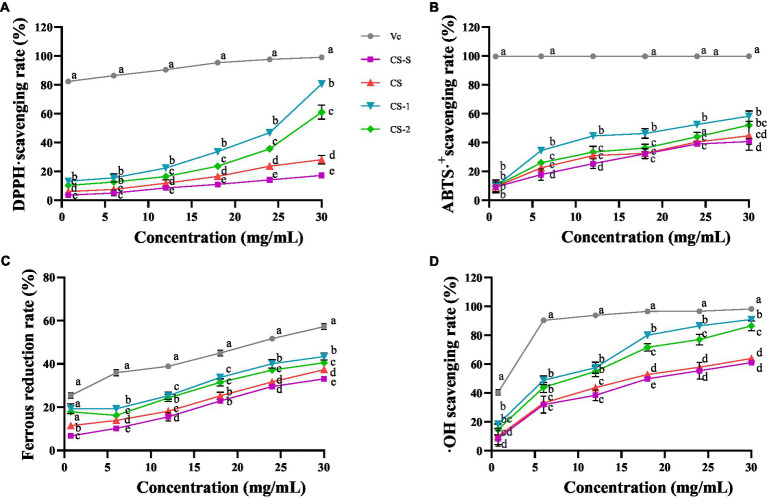
Antioxidant activities of CS and its degradation components. **(A)** DPPḤ; **(B)** ABTS^+^·; **(C)** FRAP; **(D)** ·OH. x ± s, *n* ≥ 3, different letters indicate significant differences for each component (*p* < 0.05).

The ability of CS and its degradation components to scavenge DPPḤ free radicals is shown in Figure 2A. CS and its degradation components exhibited DPPḤ free radical scavenging ability in a concentration-dependent manner. At 30 mg/mL, CS-1 and CS-2 exhibited clearance rates of 79.92 ± 0.84% and 58.32 ± 4.85%, respectively, surpassing those of CS by factors of 1.83 and 1.07, respectively. As shown in Figure 2B, 30 mg/mL of CS-S, CS, CS-1, and CS-2 exhibited an ABTS^+^· clearance rate of 40.97 ± 0.02%, 44.62 ± 0.10%, 58.27 ± 0.03%, and 52.04 ± 0.01%, respectively. Notably, CS-1 showed a significant improvement in clearance rate for ABTS^+^· compared to CS. As shown in Figure 2C, CS and its degradation components displayed good ferrous reduction abilities, which gradually increased with increasing sample concentration. At 30 mg/mL, CS-1 exerted the strongest reduction ability, surpassing that of CS by a factor of 1.07 and that of CS-2 by 0.07. Of note, 30 mg/mL CS-S, CS, CS-1, and CS-2 significantly differed in their ability to clear ·OH, with values of 61.00 ± 1.68%, 63.85 ± 2.29%, 90.84 ± 0.35%, and 86.46 ± 3.44%, respectively (Figure 2D).

### 3.3 Effects of LMWCS on cognitive function in LPS-induced mice

**Figure 3:**
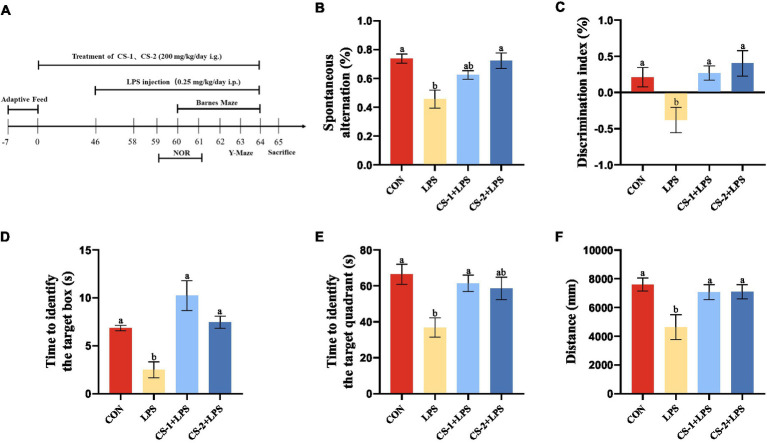
Suppression of LPS-induced learning and memory impairment by LMWCS. **(A)** Timeline for the animal experiments; **(B)** Y-maze experiment; **(C)** New object recognition experiment; **(D)** Barnes target hole exploration time; **(E)** Total distance in the Barnes target quadrant; **(F)** Barnes target quadrant exploration time. Data are presented as mean ± SEM, *n* ≥ 6, different letters indicate significant differences for each component (*p* < 0.05).

By utilizing the natural exploratory behavior of mice in novel and diverse environments, the Y-maze test can effectively measure the spatial work abilities of experimental animals. The animal time-processing axis is shown in Figure 3A. Based on the Y-maze test (Figure 3B), the percentage of spontaneous alternation in mice in the LPS group significantly decreased compared to that of mice in the CON group (*p* < 0.05), indicating a notable reduction in autonomous and working memory abilities after LPS treatment. This trend was reversed in the CS-1 and CS-2 groups (*p* < 0.05), suggesting that LMWCS can effectively improve the spatial working memory of LPS-induced mice.

During the period of the novel object recognition test, the learning and memory abilities of mice were evaluated based on their innate preference for novelty. As shown in Figure 3C, mice in the LPS group had a significant reduction in the discrimination index of new and old objects (*p* < 0.05). Compared with the LPS group, mice in both the CS-1 and CS-2 groups had a significant increase in the discrimination index of new objects (*p* < 0.05), with more evident improvement effects observed for mice in the CS-2 group.

The Barnes maze was employed to evaluate spatial learning and memory. As shown in Figure 3D, the exploration time of the Barnes target box was significantly reduced in the LPS group (*p* < 0.05). Intriguingly, mice in the CS-1 and CS-2 groups had significantly longer exploration time than mice in the LPS group (*p* < 0.05), with a duration 3.87- and 2.76-fold longer than that in the LPS group. As illustrated in Figure 3E, compared to the CON group, the Barnes target quadrant exploration time in the LPS group was significantly reduced (*p* < 0.05), and the exploration time in the CS-1 group was significantly higher than that in the LPS group (*p* < 0.05), with no significant difference found relative to the CON group. The exploration distances of the CS-1 and CS-2 groups were significantly higher than that of the LPS group (*p* < 0.05) (Figure 3F), indicating that LMWCS effectively improved spatial memory in LPS-induced mice. No significant differences were observed between the CS-1 and CS-2 interventions.

### 3.4 Effects of LMWCS on inflammatory response and oxidative stress in mouse brain

**Figure 4:**
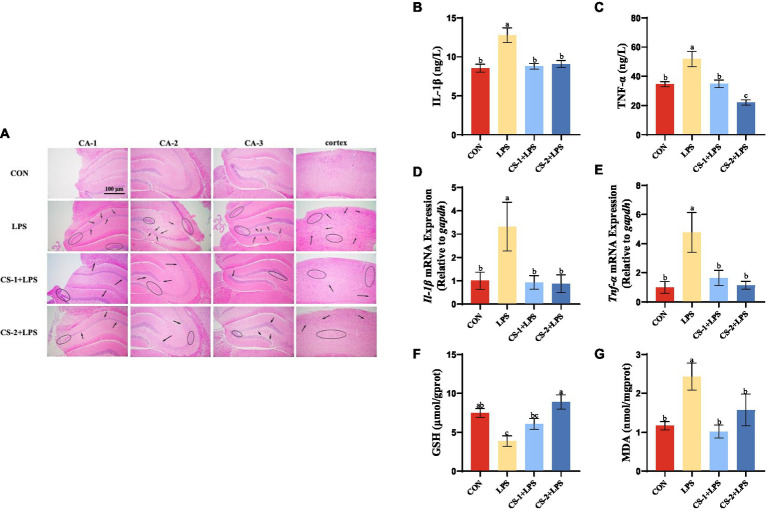
LMWCS alleviates LPS-induced mice brain inflammatory response and oxidative stress **(A)** HE staining of the cortex and hippocampus region (×200); Levels of IL-1β **(B)** and TNF-α **(C)** in serum; mRNA expressions of *Il-1β* **(D)** and *Tnf-α* **(E)** in the cortex; Levels of GSH **(F)** and MDA **(G)** in the brain. Arrows indicate the loose and disordered arrangement of neurons, and circles indicate pinpoint clear nuclear pyknosis morphological changes. Data are presented as mean ± SEM, *n* ≥ 6, different letters indicate significant differences for each component (*p* < 0.05).

To reveal the potential neuroprotective effects of LMWCS supplementation against LPS-induced damage, H&E staining was performed to observe the morphology and distribution of neuronal cells in the hippocampus and cortex of mice. As illustrated in Figure 4A, the arrangement of neurons in the LPS-induced mouse brain was loose and disordered, and neurons disappeared or underwent nuclear pyknotic morphological changes. However, LMWCS supplementation significantly ameliorated these morphological abnormalities and reduced neuronal loss. To determine the impact of LMWCS on LPS-induced systemic and brain inflammatory responses, the expressions of inflammatory mediators, such as IL-1β and TNF-α were further detected. TNF-α is one of initial factors leading to the cascade reaction of inflammatory mediators and plays a positive feedback role in amplifying inflammation (31). In addition, IL-1β is closely related to the formation of acute neuroinflammation *in vivo*. IL-1β in the brain of rodents will cause the rapid activation of astrocytes and microglia, which are the main immune cells in the brain, and their activation is the key feature of neuroinflammation (32). As depicted in Figures 4B,C, compared to the CON group, the levels of IL-1β and TNF-α significantly increased in the serum of mice in the LPS group (*p* < 0.05), while those of these inflammatory mediators in the serum of mice in the CS-1 and CS-2 groups were obviously reduced (*p* < 0.05). CS-1 and CS-2 also substantially downregulated the expressions of inflammation-related genes in mouse brain tissue, including the mRNA levels of *Il-1β* and *Tnf-α* (*p* < 0.05) (Figures 4D,E). In terms of reducing oxidative stress in the brain, as depicted in Figures 4F,G, dietary supplementation with CS-1 and CS-2 enhanced the levels of GSH in brain tissue and inhibited the production of the lipid peroxidation product, MDA (*p* < 0.05). This result indicates that LMWCS regulated the oxidative stress state, as well as dampened neuroinflammation, thereby mitigating LPS-induced learning and memory disorders in mice.

### 3.5 Effects of LMWCS on LPS-induced mice colon damage and changes in SCFAs

**Figure 5:**
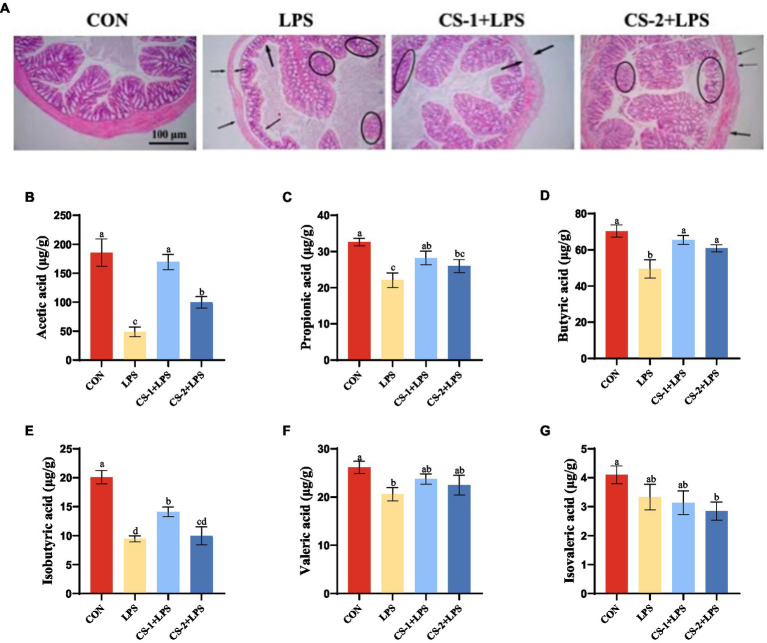
Effects of LMWCS on LPS-induced colon tissue damage and SCFAs production. **(A)** Colon tissue HE staining (×200); Levels of **(B)** Acetic acid **(C)** Propionic acid **(D)** Butyric acid **(E)** Isobutyric acid **(F)** Valeric acid **(G)** Isovaleric acid in mouse colon. Arrows indicate the loose and disordered arrangement of neurons, and circles indicate pinpoint clear nuclear pyknosis morphological changes. Data are presented as mean ± SEM, *n* ≥ 6, different letters indicate significant differences for each component.

H&E staining (Figure 5A) revealed a normal colon tissue structure in CON mice, with no signs of mucosal, crypt, or glandular damage. No evidence of inflammatory cell infiltration and abundant goblet cells was found. In contrast, the colon tissues of mice in the LPS group were disordered and characterized by mucosal and submucosal erosion, severe ulceration, bleeding, and granulation tissue formation. The crypt structure was disordered with cryptitis and crypt abscess formation. The glandular arrangement was irregular and marked by atrophy, infiltration by numerous inflammatory cells and neutrophils, and a decrease in the number of goblet cells. After LMWCS intervention, compared to the LPS group, the CS-1 and CS-2 groups showed varying degrees of improvement in lesions. Mucosal lesions were only observed in the mucosa and submucosa, with reduced damage to the crypts and glands, reduced inflammatory cells, and increased goblet cells.

The SCFA content in the intestinal microflora was determined using GC–MS, and the effects of LMWCS on LPS-induced intestinal microflora metabolism in mice were explored. As shown in Figure 5B, compared to the CON group, the acetic acid content in the colon of the LPS group was significantly reduced (*p* < 0.05). Following supplementation with CS-1 and CS-2, the acetic acid content significantly increased (*p* < 0.05). In particular, CS-1 + LPS had a better improvement effect, exhibiting a 3.88-fold higher effect than that of LPS mice. As depicted in Figure 5C, the propionic acid content in the LPS group was significantly lower than that in the CON group (*p* < 0.05). After supplementation with LMWCS, the propionic acid content in the CS-1 + LPS and CS-2 + LPS groups was significantly higher than that in the LPS group (*p* < 0.05), with no significant difference between CS-1 and CS-2. As shown in Figure 5D, the butyric acid content in the colon of mice in the CS-1 + LPS and CS-2 + LPS group was significantly higher than that of mice in the LPS group (*p* < 0.05). Surprisingly, supplementation with CS-1 increased the level of isobutyric acid (*p* < 0.05), while CS-2 did not (Figure 5E). There was no significant difference in the valeric acid and isovaleric acid content in the CS-1 + LPS and CS-2 + LPS groups compared with the LPS group (Figures 5F,G).

### 3.6 Effects of LMWCS on intestinal microbiota homeostasis in LPS-induced mice

**Figure 6:**
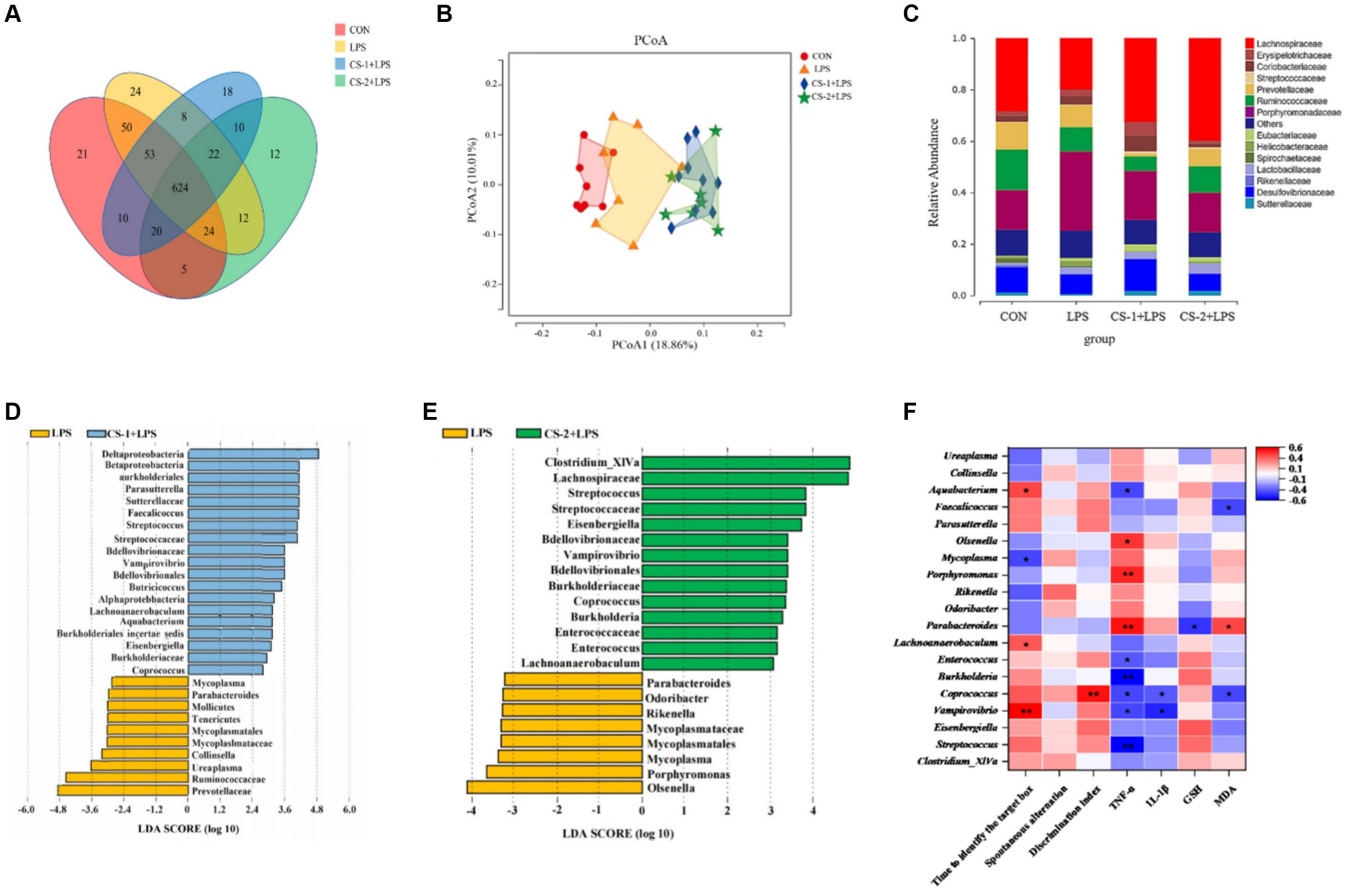
Reshaping effects of LMWCS on gut microflora in LPS-induced mice. **(A)** Venn diagram; **(B)** PCoA diagram; **(C)** Structure of the gut microbiota at the family level; **(D)** LDA scores of the differentially abundant taxa (LPS and CS-1 + LPS); **(E)** LDA scores of the differentially abundant taxa (LPS and CS-2 + LPS); **(F)** Correlation analysis. Colors of squares represent the R-value of Spearman’s correlation. ★ and ★★ indicate the significance of association at levels of *p* < 0.05 and *p* < 0.01, respectively.

The Venn plot was generated to analyze the similarity and overlap in OTU composition among the different treatment groups. All groups had the same 624 OTUs. The unique OTU numbers in the CON, LPS, CS-1 + LPS, and CS-2 + LPS groups were 21, 24, 18, and 12, respectively (Figure 6A). Therefore, treatment with CS-1 and CS-2 can significantly affect the OTU composition of the intestinal microbiota in mice altered by LPS treatment. As shown in the Principal Coordinate Analysis (PCoA) diagram (Figure 6B), the gut microbiota structure significantly differed among the CON, LPS, CS-1, and CS-2 groups, indicating that dietary supplementation with LMWCS led to alterations in the β-diversity of intestinal microorganisms in mice. The changes in the composition of the gut microbiota at the family level in each group are shown in Figure 6C. At the family level, the gut microbiota is mainly composed of *Lachnospiraceae*, *Erysipelotrichaceae*, *Coriobacteriaceae*, *Ruminococcaceae*, *Porphyromonadaceae*, and *Desulfovibrionaceae*. The mice in the LPS group had markedly increased enrichment of *Porphyromonadaceae*, *Erysipelotrichaceae*, and *Coriobacteriaceae* (associated with gastrointestinal disease) and decreased relative abundance of *Lacnospiraceae* and *Ruminococcaceae* (SCFAs-producing) compared to the control group, while the change that was restored by the LMWCS treatments. Compared to treatment with LPS, dietary supplementation with LMWCS significantly increased the relative abundances of *Sutterellaceae*, *Streptococcaceae*, *Eubacteriaceae,* and *Lachnospiraceae*, while reducing the relative abundance of *Erysipelotrichaceae* and *Porphyromonaceae*, indicating that CS-1 and CS-2 can maintain the steady-state of the microbiota to varying degrees. Furthermore, the abundances of *Streptococcus*, *Eisenbergiella*, *Vampirovibrio*, *Coprococcus*, *Enterococcus* and *Lachnoanaerobaculum* were significantly increased (*p* < 0.05) in the intestines of mice in the CS-1 and CS-2 group, whereas those of *Parabacteroides* and *Mycoplasma* were significantly decreased (*p* < 0.05) (Figures 6D,E).

To assess the potential relationship between the distinct bacteria and amelioration of LPS-induced cognitive deficits in mice administered CS-1 and CS-2, a correlation analysis was performed based on the Spearman correlation coefficient. As shown in Figure 6F, *Parabacteroides*, the dominant genus in LPS group showed a negative correlation with GSH content but a positive correlation with MDA content. *Streptococcus*, *Vampirovibrio*, *Burkholderia*, *Enterococcus* and *Aquabacterium* were negatively associated with serum TNF-α levels, while *Parabacteroides*, *Porphyromonas* and *Olsenella* showed the positive correlation. *Vampirovibrio*, *Coprobacillus*, *Lachnoanaerobaculum* and *Aquabacterium* showed positive correlations with Barnes target hole exploration time, while *Mycoplasma* was negatively associated. These results indicate that CS-1 and CS-2 alleviate LPS-induced learning and cognitive impairment in mice by regulating the gut microbiota.

## 4 Discussion and conclusion

Beef is an important, nutrient-rich livestock product. The annual slaughter volume of raw cattle in China exceeds 100 million, resulting in substantial by-products, such as beef cartilage. Shaanxi’s cattle farming industry has a long history with obvious regional characteristics and abundant genetic resources. Yellow cattle account for approximately 80% of the cattle population. Shaanxi yellow cattle are associated with several advantages, such as tolerance to rough feeding, strong resistance to stress, and good meat quality. However, the current situation is characterized by low resource utilization, high waste, low processing efficiency, and limited value addition in the beef cattle industry. Therefore, in this study, cost-effective and sustainable Shaanxi agricultural and animal husbandry waste, specifically Shaanxi yellow cattle nasal cartilage, was employed as the main raw material to “turn waste into treasure,” and the degradation of extracted and purified CS was controlled to obtain LMWCS (14.7 kDa CS-1 and 50.9 kDa CS-2). Based on the results, supplementation with CS-1 and CS-2 significantly increased the percentage of spontaneous alternation in the Y-maze, the new object recognition index in the novel object recognition test, and the Barnes target region exploration time (*p* < 0.05). Therefore, LMWCS improves LPS-induced cognitive impairment in mice. Our findings are of utmost importance for improving the utilization of agricultural and animal husbandry resources, filling market gaps, enhancing market competitiveness, and developing local economies.

Currently, various methods, including enzymatic (33), physical (26, 34), and chemical degradation, are commonly used to prepare low-molecular-weight polysaccharides through degradation (35–37). Using a physical degradation method to prepare low-molecular-weight samples is straightforward and leads to minimal environmental pollution from chemical waste. However, the yield is low. Enzymatic degradation is associated with high specificity and efficiency. Thus, identifying suitable glycosidase-active hydrolases for the specific preparation of oligosaccharides has become a hotspot in research. The most used degradation enzymes are commercially available but are expensive. Although the acid degradation method for preparing low-molecular-weight polysaccharides is associated with poor specificity, it offers complete degradation, high efficiency, and simple operation and has wide applications in the extraction, depolymerization, and mass spectrometry analysis of polysaccharides. The H_2_O_2_ degradation method, is also known as the oxidative degradation method or the free radical degradation method. H_2_O_2_ is unstable, and its residues in food processing will be decomposed soon, so it is allowed to be used in the food processing, such as *Tremella fuciformis*, *Sargassum fusiforme* and *Codium cylindricum* (38). In this system, oxygen radicals are produced by H_2_O_2_, which has unpaired electrons and high chemical reactivity for degrading polysaccharides by breaking down polysaccharide chains. Vc can provide electrons to HO or O^2 −^, rendering them inactive. However, Vc can also generate H_2_O_2_ or HO^−^ through self-oxidation (39). When the molecular weight is large, the polysaccharide chains are long or have many branches, and radicals received within a certain amount of time have strong action, more cutting points, and large changes in the amplitude of their molecular weight. However, when the molecular weight is relatively small, the sugar chain shortens, and the action of the received free radicals within a certain amount of time is smaller, with fewer cut points, and the extent of molecular weight change is small. These introduce complexity to the system that requires careful management to optimize the degradation process and minimize unintended oxidative reactions.

The molecular weight and chain length of polysaccharides are crucial structural attributes that dictate their pharmacological effects. The molecular weight indirectly affects their physicochemical properties such as solubility and viscosity, thus affecting the absorption of polysaccharide in the body. Polysaccharides with large molecular weight are difficult to cross the cell membrane and enter the organism, while polysaccharides with too small molecular weight are difficult to form an active spatial structure (40). By using ultrasound to degrade *Ganoderma lucidum* polysaccharides, Xu et al. found that as the molecular weight of *Ganoderma lucidum* polysaccharides decreased from 3.06 × 10^3^ kDa to 13.6 kDa, the small molecular weight *Ganoderma lucidum* polysaccharide fragment exhibited better effects at reducing the content of MDA in mice and increasing the activity of glutathione peroxidase and superoxide dismutase in mouse serum than *Ganoderma lucidum* polysaccharide (41). Zhou et al. purified α-1,6-galactan from Cantharellus cibarius *Fr.* (chanterelle) and reduced its molecular weight from approximately 20 kDa to 5 kDa (named CCP) to increase its solubility and absorption. In APP/PS1 mice, CCP improved both spatial and non-spatial memory loss in AD mice, as confirmed by the Morris water maze, step-down, step-through, and novel object recognition tests, and dampened the deposition of amyloid-β plaques (42). In this study, CS-1 and CS-2 obtained from the H_2_O_2_-Vc degradation system showed no significant changes in the sulfate and uronic acid contents of the extracted CS. According to the infrared spectrum detection results, degradation did not damage the main structure of the CS. Although the results suggest that both CS-1 and CS-2 exhibit inhibitory effects on neuroinflammation and improve cognitive impairment, CS-1 had stronger free radical scavenging abilities and promoted the production of SCFAs, such as acetic acid and isobutyric acid. In contrast, CS-2 led to a greater downregulation of the inflammatory factor, TNF-α, and upregulation of brain GSH levels.

In addition to improving oxidative stress and inflammatory responses, maintaining intestinal microbiota homeostasis plays an important role in cognitive interventions. Over the past decade, numerous studies have revealed that environmental factors affect the composition of the gut microbiome, which significantly impacts host metabolism through specific interactions with the host (43). Dysbiosis of the gut microbiota can accelerate cognitive impairment. Normally, the gut microbiota remains relatively stable over extended periods. However, factors, including aging, dietary choices, antibiotic usage, and low physical activity, can disrupt the diversity of gut microbiota, resulting in dysbiosis, which may contribute to the development of various age-related conditions associated with cognitive impairment, including Alzheimer’s disease, amnesia, mild cognitive impairment, etc. (44). In our study, *Porphyromonaceae* and *Erysipelotrichaceae* significantly increased in the LPS-treated group, whereas the relative abundance of beneficial bacteria, such as *Lachnospiraceae* and *Ruminococcaceae*, significantly decreased at the family level. Recent studies found a notable increase in *Erysipelotrichaceae* among patients with AD in China (45). Furthermore, compared to the healthy population, the abundance of *Lachnospiraceae* in the gut microbiota of patients with AD was found to decrease (46). *Lachnospiraceae* is known for its robust ability to produce short-chain fatty acids (SCFAs), such as butyrate. SCFAs are produced in the rectum and colon through fermentation of dietary fiber by gut microbiota (47). Valeric, isovaleric, butyric, isobutyric, propionic, and acetic acids can interfere with the activation of microglia and astrocytes, reduce inflammatory reactions, and reduce tau protein and Aβ sedimentation. Compared with the LPS group, dietary supplementation with CS-1 and CS-2 significantly increased the relative abundance of *Sutterellaceae*, *Streptococcaceae*, and *Lachnospiraceae*, and decreased that of *Erysipelotrichaceae* and *Porphyromonaceae* in this experimental study. Supplementation with CS-1 and CS-2 also increased the levels of SCFAs, such as acetic, propionic, and butyric acids.

Different molecular weight polysaccharides differ in regulating the intestinal flora. Kong et al. found that low molecular weight seaweed sulfate polysaccharide (<30 kDa) showed better prebiotic activities than high molecular weight sulfate polysaccharide (> 30 kDa) in increasing the abundance of *Lactobacillus* and *Bifidobacterium*, and SCFAs levels (48). Guar gum, a water-soluble polysaccharide, has been studied, and findings indicate that hydrolyzed guar gum with molecular weights of 10 and 15 kDa yields the highest proportion of butyrate (49). Chitosans (Mw ≤ 200 kDa) could increase the diversity of DSS-induced UC mice intestinal flora better than chitosans (Mw ≤ 50 kDa) and chitosans (Mw ≤ 3 kDa), especially in promoting the relative abundance of *Prevotellaceae_UCG-001* (50). Liu et al. found that there were differences in the composition of intestinal microbiota in obese mice after dietary supplementation with different molecular weights of konjac glucomannan (KGM): the abundance of *Porphyromonadaceae*, *Ruminococcaceae*, *Verrucomicrobiaceae*, *Prevotellaceae* and *Helicobacteraceae* were increased significantly after 90 kDa KGM supplementation; while, when mice were fed with 5 kDa KGM, the abundance of *Erysipelotrichaceae*, *Lachnospiraceae*, *Helicobacteraceae* and *Bifidobacteriaceae* were obviously up-regulated (51). In the present study, *Coprococcus* was found to belong to the dominant microbial flora at the genus levels in mice treated with CS-1 and CS-2. *Coprococcus* is an important member of the *Firmicutes*, *Pilspirillaceae*, and an important genus in the gut. They are mostly isolated from feces, actively ferage carbohydrates, and are one of the important producers of butyrate (52). Butyrate is a short-chain fatty acid with a variety of biological activities, including anti-inflammatory effects. Thus, by producing butyrate, *coprococcus* may, to some extent, have anti-inflammatory properties capable of reducing or relieving inflammation. In addition, *Coprococcus* has been used as a microbial biomarker to assess the health of the human gastrointestinal tract. It has been suggested that bacteria of the genus *Coprococcus* may help to suppress immune responses and reduce the severity of allergic reactions. These properties further support the idea that *Coprococcus* has anti-inflammatory effects. *Mycoplasma* is a kind of prokaryotic cell type microorganism, which widely exists in humans and animals (53). Some of them are pathogenic to humans, such as *Mycoplasma pneumoniae* and *Ureaplasma urealyticum*. These pathogenic *mycoplasmas* can cause a variety of infections, including pneumonia, bronchitis, etc., which are often accompanied by inflammatory processes. For example, mycoplasma pneumonia, which is caused by *Mycoplasma pneumoniae* infection, has clinical manifestations that include pulmonary inflammation with refractory and violent cough. However, further exploration is warranted to elucidate the specific mechanism, particularly the relationship between the complex structures of CS and LMWCS and their regulation of intestinal microecology. In summary, LMWCS (14.7 kDa CS-1 and 50.9 kDa CS-2), specifically low molecular weight CS-1 derived from the controlled degradation of bovine nasal cartilage, can mitigate LPS-induced oxidative stress and inflammatory responses in the mouse brain, and improve intestinal microbiota homeostasis, suggesting its significance as a key component for cognitive intervention. These findings highlight the potential applications of low-molecular-weight chondroitin sulfate in brain nutrition products.

## Data availability statement

The data presented in the study are deposited in the https://www.ncbi.nlm.nih.gov/, repository, accession number PRJNA1075476.

## Ethics statement

The animal study was approved by the Animal Ethics Committee of Northwest A&F University. The study was conducted in accordance with the local legislation and institutional requirements.

## Author contributions

YD: Data curation, Formal analysis, Investigation, Methodology, Project administration, Software, Validation, Visualization, Writing – original draft. QinL: Data curation, Formal analysis, Investigation, Methodology, Software, Validation, Visualization, Writing – original draft. JS: Data curation, Investigation, Validation, Writing – original draft. RG: Project administration, Supervision, Writing – original draft. TM: Software, Visualization, Writing – original draft. ZL: Project administration, Resources, Supervision, Writing – review & editing. QiaL: Funding acquisition, Project administration, Resources, Supervision, Writing – review & editing.

## References

[1] Sun Y Koyama Y Shimada S. Inflammation from peripheral organs to the brain: how does systemic inflammation cause Neuroinflammation? Front Aging Neurosci. (2022) 14:903455. doi: 10.3389/fnagi.2022.903455, PMID: 35783147 PMC9244793

[2] Huang X Hussain B Chang J. Peripheral inflammation and blood-brain barrier disruption: effects and mechanisms. CNS Neurosci Ther. (2021) 27:36–47. doi: 10.1111/cns.13569, PMID: 33381913 PMC7804893

[3] Teleanu DM Niculescu AG Lungu II Radu CI Vladacenco O Roza E. An overview of oxidative stress, neuroinflammation, and neurodegenerative diseases. Int J Mol Sci. (2022) 23:11. doi: 10.3390/ijms23115938, PMID: 35682615 PMC9180653

[4] Low A Mak E Malpetti M Passamonti L Nicastro N Stefaniak JD. In vivo neuroinflammation and cerebral small vessel disease in mild cognitive impairment and Alzheimer's disease. J Neurol Neurosurg Psychiatry. (2020) 92:45–52. doi: 10.1136/jnnp-2020-323894, PMID: 32917821 PMC7803899

[5] Troubat R Barone P Leman S Desmidt T Cressant A Atanasova B. Neuroinflammation and depression: a review. Eur J Neurosci. (2021) 53:151–71. doi: 10.1111/ejn.14720 32150310

[6] Hey GE Vedam-Mai V Beke M Amaris MA Ramirez-Zamora A. The Interface between inflammatory bowel disease, Neuroinflammation, and neurological disorders. Semin Neurol. (2023) 43:572–82. doi: 10.1055/s-0043-1771467, PMID: 37562450

[7] Ding J-H Jin Z Yang X-X Lou J Shan W-X Hu Y-X. Role of gut microbiota via the gut-liver-brain axis in digestive diseases. World J Gastroenterol. (2020) 26:6141–62. doi: 10.3748/wjg.v26.i40.6141 33177790 PMC7596643

[8] Vogt NM Kerby RL Dill-Mcfarland KA Harding SJ Merluzzi AP Johnson SC. Gut microbiome alterations in Alzheimer's disease. Sci Rep. (2017) 7:1P563. doi: 10.1038/s41598-017-13601-y, PMID: 29051531 PMC5648830

[9] Cattaneo A Cattane N Galluzzi S Provasi S Lopizzo N Festari C. Association of brain amyloidosis with pro-inflammatory gut bacterial taxa and peripheral inflammation markers in cognitively impaired elderly. Neurobiol Aging. (2017) 49:60–8. doi: 10.1016/j.neurobiolaging.2016.08.019, PMID: 27776263

[10] Lee JW Lee YK Yuk DY Choi DY Ban SB Oh KW. Neuro-inflammation induced by lipopolysaccharide causes cognitive impairment through enhancement of beta-amyloid generation. J Neuroinflammation. (2008) 5:137. doi: 10.1186/1742-2094-5-37, PMID: 18759972 PMC2556656

[11] Ju C Wang Y Zang C Liu H Yuan F Ning J. Inhibition of Dyrk1A attenuates LPS-induced Neuroinflammation via the TLR4/NF-κB P65 signaling pathway. Inflammation. (2022) 45:2375–87. doi: 10.1007/s10753-022-01699-w, PMID: 35917097

[12] Kim AR Lim YK Kook JK Bak EJ Yoo YJ. Lipopolysaccharides of fusobacterium nucleatum and *Porphyromonas gingivalis* increase RANKL-expressing neutrophils in air pouches of mice. Lab Anim Res. (2021) 37:5. doi: 10.1186/s42826-020-00080-y, PMID: 33407938 PMC7789191

[13] Awofiranye AE Hudson J Tithi AD Linhardt RJ Vongsangnak W Koffas MAG. Chondroitin sulfate and its derivatives: a review of microbial and other production methods. Fermentation. (2022) 8:7323. doi: 10.3390/fermentation8070323

[14] Krylov VB Grachev AA Ustyuzhanina NE Ushakova NA Preobrazhenskaya ME Kozlova NI. Preliminary structural characterization, anti-inflammatory and anticoagulant activities of chondroitin sulfates from marine fish cartilage. Russ Chem Bull. (2011) 60:746–53. doi: 10.1007/s11172-011-0115-x

[15] Zhang J Zhang J Zhu C Jiang Y Shen Z Jiang X. Chondroitin sulfate oligosaccharides prepared by chondroitinase and its antioxidant activities. Sci Technol Food Ind. (2017) 38:1348–52. doi: 10.13386/j.issn1002-0306.2017.13.009

[16] Chen S Chen W Chen Y Mo X Fan C. Chondroitin sulfate modified 3D porous electrospun nanofiber scaffolds promote cartilage regeneration. Mater Sci Eng C. (2021) 118:111312. doi: 10.1016/j.msec.2020.111312, PMID: 33254957

[17] Qi SS Shao ML Sun Z Chen SM Hu YJ Li XS. Chondroitin sulfate alleviates diabetic osteoporosis and repairs bone microstructure via anti-oxidation, anti-inflammation, and regulating bone metabolism. Front Endocrinol (Lausanne). (2021) 12:759843. doi: 10.3389/fendo.2021.759843, PMID: 34777254 PMC8579055

[18] Baici A Horler D Moser B Hofer HO Fehr K Wagenhauser FJ. Analysis of glycosaminoglycans in human serum after oral administration of chondroitin sulfate. Rheumatol Int. (1992) 12:81–8. doi: 10.1007/BF00290259, PMID: 1411092

[19] Zou ZH Wei M Fang J Dai W Sun TT Liu Q. Preparation of chondroitin sulfates with different molecular weights from bovine nasal cartilage and their antioxidant activities. Int J Biol Macromol. (2020) 152:1047–55. doi: 10.1016/j.ijbiomac.2019.10.192, PMID: 31751707

[20] Ji X Guo J Ding D Gao J Hao L Guo X. Structural characterization and antioxidant activity of a novel high-molecular-weight polysaccharide from *Ziziphus Jujuba* cv. Muzao. J Food Meas Charact. (2022) 16:2191–200. doi: 10.1007/s11694-022-01288-3

[21] Liu W Wang HY Pang XB Yao WB Gao XD. Characterization and antioxidant activity of two low-molecular-weight polysaccharides purified from the fruiting bodies of Ganoderma lucidum. Int J Biol Macromol. (2010) 46:451–7. doi: 10.1016/j.ijbiomac.2010.02.006, PMID: 20153767

[22] Wang K Qi L Zhao L Liu J Guo Y Zhang C. Degradation of chondroitin sulfate: mechanism of degradation, influence factors, structure-bioactivity relationship and application. Carbohydr Polym. (2023) 301:120361. doi: 10.1016/j.carbpol.2022.120361, PMID: 36446498

[23] Yuan L Chu Q Wu X Yang B Zhang W Jin W. Anti-inflammatory and antioxidant activity of peptides from ethanol-soluble hydrolysates of sturgeon (*Acipenser schrenckii*) cartilage. Front Nutr. (2021) 8:689648. doi: 10.3389/fnut.2021.689648, PMID: 34179062 PMC8225940

[24] Yang J Shen M Wu T Chen X Wen H Xie J. Physicochemical, structural characterization, and antioxidant activities of chondroitin sulfate from *Oreochromis niloticus* bones. Food Sci Human Wellness. (2023) 12:1102–8. doi: 10.1016/j.fshw.2022.10.027

[25] Unver T Erenler AS Bingul M Boga M. Comparative analysis of antioxidant, anticholinesterase, and antibacterial activity of microbial chondroitin sulfate and commercial chondroitin sulfate. Chem Biodivers. (2023) 20:10e202300924. doi: 10.1002/cbdv.202300924 37615364

[26] Liu Z Chen Y Qiao Q Sun Y Liu Q Ren B. Sesamol supplementation prevents systemic inflammation-induced memory impairment and amyloidogenesis via inhibition of nuclear factor kappaB. Mol Nutr Food Res. (2017) 61:1600734. doi: 10.1002/mnfr.201600734, PMID: 27860258

[27] Liu Q Chen Y Shen C Xiao Y Wang Y Liu Z. Chicoric acid supplementation prevents systemic inflammation-induced memory impairment and amyloidogenesis via inhibition of NF-kappaB. FASEB J. (2017) 31:1494–507. doi: 10.1096/fj.201601071R, PMID: 28003341

[28] Wang Y Zhang Y Wang X Li Q Zhao Y Jiang Y. Sesamol mitigates chronic Iron overload-induced cognitive impairment and systemic inflammation via IL-6 and DMT1 regulation. Mol Nutr Food Res. (2023) 67:17e2300012. doi: 10.1002/mnfr.202300012 37452409

[29] Liu Q Xi Y Wang Q Liu J Li P Meng X. Mannan oligosaccharide attenuates cognitive and behavioral disorders in the 5xFAD Alzheimer's disease mouse model via regulating the gut microbiota-brain axis. Brain Behav Immun. (2021) 95:330–43. doi: 10.1016/j.bbi.2021.04.005, PMID: 33839232

[30] Garnjanagoonchorn W Wongekalak L Engkagul A. Determination of chondroitin sulfate from different sources of cartilage. Chem Eng Process. (2007) 46:465–71. doi: 10.1016/j.cep.2006.05.019

[31] Singh A Ansari VA Mahmood T Ahsan F Wasim R. Neurodegeneration: microglia: Nf-Kappab signaling pathways. Drug Res (Stuttg). (2022) 72:496–9. doi: 10.1055/a-1915-4861 36055286

[32] Almeida PGC Nani JV Oses JP Brietzke E Hayashi MAF. Neuroinflammation and glial cell activation in mental disorders. Brain Behav Immun Health. (2020) 2:100034. doi: 10.1016/j.bbih.2019.100034, PMID: 38377429 PMC8474594

[33] Wang J Li L Wang Z Cui Y Tan X Yuan T. Supplementation of lycopene attenuates lipopolysaccharide-induced amyloidogenesis and cognitive impairments via mediating neuroinflammation and oxidative stress. J Nutr Biochem. (2018) 56:16–25. doi: 10.1016/j.jnutbio.2018.01.009, PMID: 29454265

[34] Diao Z Li J Liu Q Wang Y. In-vivo metabolite profiling of chicoric acid in rat plasma, urine and feces after oral administration using liquid chromatography quadrupole time of flight mass spectrometry. J Chromatogr B Analyt Technol Biomed Life Sci. (2018) 1081–1082:8–14. doi: 10.1016/j.jchromb.2018.02.016 29494984

[35] Song G Liu Z Liu Q Liu X. Lipoic acid prevents acrylamide-induced neurotoxicitys in CD-1 mice and BV2 microglial cells via maintaining redox homeostasis. J Funct Foods. (2017) 35:363–75. doi: 10.1016/j.jff.2017.05.058

[36] Liu ZG Sun YL Qiao QL Zhao T Zhang WT Ren B. Sesamol ameliorates high-fat and high-fructose induced cognitive defects via improving insulin signaling disruption in the central nervous system. Food Funct. (2017) 8:710–9. doi: 10.1039/c6fo01562j, PMID: 28102395

[37] Wang Y Diao Z Li J Ren B Zhu D Liu Q. Chicoric acid supplementation ameliorates cognitive impairment induced by oxidative stress via promotion of antioxidant defense system. RSC Adv. (2017) 7:36149–62. doi: 10.1039/c7ra06325c

[38] Chen X Li X Sun-Waterhouse D Zhu B You L Hileuskaya K. Polysaccharides from Sargassum fusiforme after UV/H(2)O(2) degradation effectively ameliorate dextran sulfate sodium-induced colitis. Food Funct. (2021) 12:11747–59. doi: 10.1039/d1fo02708e 34806724

[39] Ofoedu C You L Osuji C Iwouno JO Korzeniowska M. Hydrogen peroxide effects on natural-sourced polysaccharides: free radical formation/production, degradation process, and reaction mechanism – a critical synopsis. Food Secur. (2021) 10:4699. doi: 10.3390/foods10040699, PMID: 33806060 PMC8064442

[40] Wang B Yan L Guo S Wen L Yu M Feng L. Structural elucidation, modification, and structure-activity relationship of polysaccharides in Chinese herbs: a review. Front Nutr. (2022) 9:908175. doi: 10.3389/fnut.2022.908175, PMID: 35669078 PMC9163837

[41] Xu Y Zhang X Yan X-H Zhang J-L Wang L-Y Xue H. Characterization, hypolipidemic and antioxidant activities of degraded polysaccharides from Ganoderma lucidum. Int J Biol Macromol. (2019) 135:706–16. doi: 10.1016/j.ijbiomac.2019.05.166, PMID: 31129213

[42] Zhou A Cheng H Liu H Li L Chen Z Chen S. Neuroprotection of low-molecular-weight galactan obtained from Cantharellus cibarius Fr. Against Alzheimer's disease. Carbohydr Polym. (2023) 316:121033. doi: 10.1016/j.carbpol.2023.121033, PMID: 37321728

[43] Rubert J Schweiger PJ Mattivi F Tuohy K Jensen KB Lunardi A. Intestinal organoids: a tool for modelling diet-microbiome-host interactions. Trends Endocrinol Metab. (2020) 31:848–58. doi: 10.1016/j.tem.2020.02.004, PMID: 33086077

[44] Dong S Sun M He C Cheng H. Brain-gut-microbiota axis in Parkinson's disease: a historical review and future perspective. Brain Res Bull. (2022) 183:84–93. doi: 10.1016/j.brainresbull.2022.02.015, PMID: 35245613

[45] Zheng S Pan L Hou J Liao A Hou Y Yu G. The role of wheat embryo globulin nutrients in improving cognitive dysfunction in AD rats. Food Funct. (2022) 13:9856–67. doi: 10.1039/d2fo00815g, PMID: 36047913

[46] Kaiyrlykyzy A Kozhakhmetov S Babenko D Zholdasbekova G Alzhanova D Olzhayev F. Study of gut microbiota alterations in Alzheimer's dementia patients from Kazakhstan. Sci Rep. (2022) 12:115115:15115. doi: 10.1038/s41598-022-19393-0, PMID: 36068280 PMC9448737

[47] Watanabe K Yamano M Masujima Y Ohue-Kitano R Kimura I. Curdlan intake changes gut microbial composition, short-chain fatty acid production, and bile acid transformation in mice. Biochem Biophys Rep. (2021) 27:101095. doi: 10.1016/j.bbrep.2021.101095, PMID: 34401531 PMC8358642

[48] Kong Q Dong S Gao J Jiang C. In vitro fermentation of sulfated polysaccharides from E. Prolifera and *L. japonica* by human fecal microbiota. Int J Biol Macromol. (2016) 91:867–71. doi: 10.1016/j.ijbiomac.2016.06.036, PMID: 27316763

[49] Pylkas AM Juneja LR Slavin JL. Comparison of different fibers for in vitro production of short chain fatty acids by intestinal microflora. J Med Food. (2005) 8:113–6. doi: 10.1089/jmf.2005.8.113 15857221

[50] Niu W Dong Y Fu Z Lv J Wang L Zhang Z. Effects of molecular weight of chitosan on anti-inflammatory activity and modulation of intestinal microflora in an ulcerative colitis model. Int J Biol Macromol. (2021) 193:1927–36. doi: 10.1016/j.ijbiomac.2021.11.024 34748786

[51] Liu Q Fang J Huang W Liu S Zhang X Gong G. The intervention effects of konjac glucomannan with different molecular weights on high-fat and high-fructose diet-fed obese mice based on the regulation of gut microbiota. Food Res Int. (2023) 165:112498. doi: 10.1016/j.foodres.2023.112498, PMID: 36869507

[52] Yang R Shan S Shi J Li H An N Li S. *Coprococcus eutactus*, a potent probiotic, alleviates colitis via acetate-mediated IgA response and microbiota restoration. J Agric Food Chem. (2023) 71:3273–84. doi: 10.1021/acs.jafc.2c06697 36786768

[53] Gnanadurai R Fifer H. *Mycoplasma genitalium*: a review. Microbiology (Reading). (2020) 166:21–9. doi: 10.1099/mic.0.000830 31329090

