# Supplementary material for: Intervention effects of low-molecular-weight chondroitin sulfate from the nasal cartilage of yellow cattle on lipopolysaccharide-induced behavioral disorders: regulation of the microbiome-gut-brain axis
Source: Front Nutr. 2024 May 21;11:1371691. doi: 10.3389/fnut.2024.1371691 (PMC11148680; doi:10.3389/fnut.2024.1371691)
Supplement: Supplementary file 1 [file Image_1.pdf]

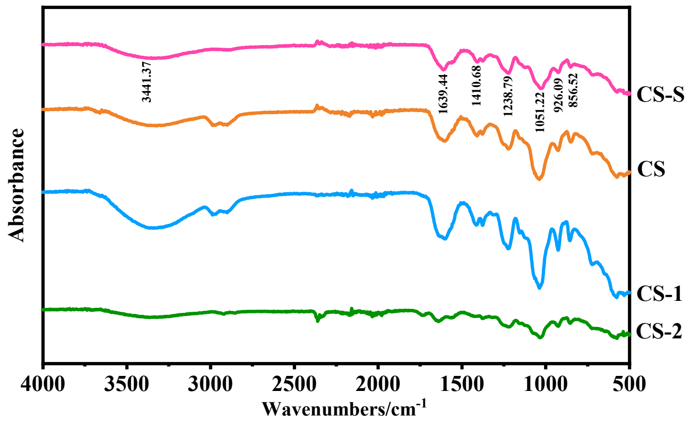

Supplementary Figure 1. Fourier transform infrared spectroscopy of CS and its degradation components
